# Not Just a Theory—The Utility of Mathematical Models in Evolutionary Biology

**DOI:** 10.1371/journal.pbio.1002017

**Published:** 2014-12-09

**Authors:** Maria R. Servedio, Yaniv Brandvain, Sumit Dhole, Courtney L. Fitzpatrick, Emma E. Goldberg, Caitlin A. Stern, Jeremy Van Cleve, D. Justin Yeh

**Affiliations:** 1 Department of Biology, University of North Carolina, Chapel Hill, North Carolina, United States of America; 2 Department of Plant Biology, University of Minnesota, Twin Cities, St. Paul, Minnesota, United States of America; 3 National Evolutionary Synthesis Center (NESCent), Durham, North Carolina, United States of America; 4 Department of Ecology, Evolution, and Behavior, University of Minnesota, Twin Cities, St. Paul, Minnesota, United States of America; 5 Santa Fe Institute, Santa Fe, New Mexico, United States of America; 6 Department of Biology, University of Kentucky, Lexington, Kentucky, United States of America

## Abstract

Models have made numerous contributions to evolutionary biology, but misunderstandings persist regarding their purpose. By formally testing the logic of verbal hypotheses, proof-of-concept models clarify thinking, uncover hidden assumptions, and spur new directions of study. thumbnail image credit: modified from the Biodiversity Heritage Library

## Summary

Progress in science often begins with verbal hypotheses meant to explain why certain biological phenomena exist. An important purpose of mathematical models in evolutionary research, as in many other fields, is to act as “proof-of-concept” tests of the logic in verbal explanations, paralleling the way in which empirical data are used to test hypotheses. Because not all subfields of biology use mathematics for this purpose, misunderstandings of the function of proof-of-concept modeling are common. In the hope of facilitating communication, we discuss the role of proof-of-concept modeling in evolutionary biology.

## A Conceptual Gap: Models and Misconceptions

Recent advances in many fields of biology have been driven by a synergistic approach involving observation, experiment, and mathematical modeling (see, e.g., [1]). Evolutionary biology has long required this approach, due in part to the complexity of population-level processes and to the long time scales over which evolutionary processes occur. Indeed, the “modern evolutionary synthesis” of the 1930s and 40s—a pivotal moment of intellectual convergence that first reconciled Mendelian genetics and gene frequency change with natural selection—hinged on elegant mathematical work by RA Fisher, Sewall Wright, and JBS Haldane. Formal (i.e., mathematical) evolutionary theory has continued to mature; models can now describe how evolutionary change is shaped by genome-scale properties such as linkage and epistasis [2],[3], complex demographic variability [4], environmental variability [5], and individual and social behavior [6],[7] within and between species.

Despite their integral role in evolutionary biology, the purpose of certain types of mathematical models is often questioned [8]. Some view models as useful only insofar as they generate immediately testable quantitative predictions [9], and others see them as tools to elaborate empirically-derived biological patterns but not to independently make substantial new advances [10]. Doubts about the utility of mathematical models are not limited to present day studies of evolution—indeed, this is a topic of discussion in many fields including ecology [11],[12], physics [13], and economics [14], and has been debated in evolution previously [15]. We believe that skepticism about the value of mathematical models in the field of evolution stems from a common misunderstanding regarding the goals of particular types of models. While the connection between empiricism and some forms of theory (e.g., the construction of likelihood functions for parameter inference and model choice) is straightforward, the importance of highly abstract models—which might not make immediately testable predictions—can be less evident to empiricists. The lack of a shared understanding of the purpose of these “proof-of-concept” models represents a roadblock for progress and hinders dialogue between scientists studying the same topics but using disparate approaches. This conceptual gap obstructs the stated goals of evolutionary biologists; a recent survey of evolutionary biologists and ecologists reveals that the community wants more interaction between theoretical and empirical research than is currently perceived to occur [16].

To promote this interaction, we clarify the role of mathematical models in evolutionary biology. First, we briefly describe how models fall along a continuum from those designed for quantitative prediction to abstract models of biological processes. Then, we highlight the unique utility of proof-of-concept models, at the far end of this continuum of abstraction, presenting several examples. We stress that the development of rigorous analytical theory with proof-of-concept models is itself a test of verbal hypotheses [11],[17], and can in fact be as strong a test as an elegant experiment.

## Degrees of Abstraction in Evolutionary Theory

Good evolutionary theory always derives its motivation from the natural world and relates its conclusions back to biological questions. Building such theory requires different degrees of biological abstraction depending on the specific question. Some questions are best addressed by building models to interface directly with data. For example, DNA substitution models in molecular evolution can be built to take into account the biochemistry of DNA, including variation in guanine and cytosine (GC) content [18] and the structure of the genetic code [19]. These substitution models form the basis of the likelihood functions used to infer phylogenetic relationships from sequence data. Models can also provide baseline expectations against which to compare empirical observations (e.g., coalescent genealogies under simple demographic histories [20] or levels of genetic diversity around selective sweeps [21]).

In contrast, higher degrees of abstraction are required when models are built to qualitatively, as opposed to quantitatively, describe a set of processes and their expected outcomes. Though not mathematical, verbal or pictorial models have long been used in evolutionary biology to form abstract hypotheses about processes that operate among diverse species and across vast time scales. Darwin's [22] theory of natural selection represents one such model, and many others have followed since; for example, Muller proposed that genetic recombination might evolve to prevent the buildup of deleterious mutations (“Muller's ratchet”) [23], and the “Red Queen hypothesis” proposes that coevolution between antagonistically interacting species can proceed without either species achieving a long-term increase in fitness [24]. A clear verbal model lays out explicitly which biological factors and processes it is (and is not) considering and follows a chain of logic from these initial assumptions to conclusions about how these factors interact to produce biological patterns.

However, evolutionary processes and the resulting patterns are often complex, and there is much room for error and oversight in verbal chains of logic. In fact, verbal models often derive their influence by functioning as lightning rods for debate about exactly which biological factors and processes are (or should be) under consideration and how they will interact over time. At this stage, a mathematical framing of the verbal model becomes invaluable. It is this proof-of-concept modeling on which we focus below.

## Proof-of-Concept Models: Testing Verbal Logic in Evolutionary Biology

Proof-of-concept models, used in many fields, test the validity of verbal chains of logic by laying out the specific assumptions mathematically. The results that follow from these assumptions emerge through the principles of mathematics, which reduces the possibility of logical errors at this step of the process. The appropriateness of the assumptions is critical, but once they are established, the mathematical analysis provides a precise mapping to their consequences.

**Figure 1:**
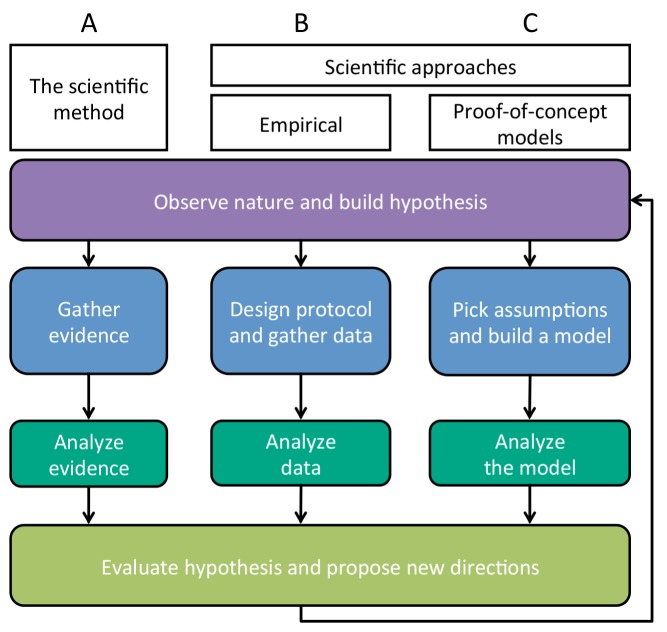
Parallels between empirical experimental techniques and proof-of-concept modeling in the scientific process. This flowchart shows the steps in the scientific process, emphasizing the relationship between experimental empirical techniques and proof-of-concept modeling. Other approaches, including ones that combine empirical and mathematical techniques, are not shown. We note that some questions are best addressed by one or the other of these techniques, while others might benefit from both approaches. Proof-of-concept models, for example, are best suited to testing the logical correctness of verbal hypotheses (i.e., whether certain assumptions actually lead to certain predictions), while only empirical approaches can address hypotheses about which assumptions are most commonly met in nature. (A) A general description of the scientific process. (B) Steps in the scientific process as approached by experimental empirical techniques. In this case, statistical techniques are often used to analyze the gathered data. (C) Steps in the scientific process as approached by proof-of-concept modeling. Here, techniques such as invasion and stability analyses, stochastic simulations, and numerical analyses are employed to analyze the expected outcomes of a model. In both cases, the hypothesis can be evaluated by comparing the results of the analyses to the original predictions.

A clear analogy exists between proof-of-concept models and other forms of hypothesis testing. In general, the hypotheses generated by verbal models must ultimately be tested as part of the scientific process (Figure 1A). Empirical research tests a hypothesis by gathering data in order to determine whether those data match predicted outcomes (Figure 1B). Proof-of-concept models function very similarly (Figure 1C): to test the validity of a verbal model, precise predictions from a mathematical analysis of the assumptions are compared against verbal predictions. This important function of mathematical modeling is commonly misunderstood, as theoreticians are often asked how they might test their proof-of-concept models empirically. The models *themselves* are tests of whether verbal models are sound; if their predictions do not match, the verbal model is flawed, and that form of the hypothesis is disproved.

That is not to say, however, that proof-of-concept models do not need to interact with natural systems or with empirical work; in fact, quite the contrary is true. There are vital links between theory and natural systems at the assumption stage (Box 1), and there can also be important connections at the predictions stage (Box 2); connections also occur at the discussion stage, where empirical results are synthesized into a broader conceptual framework. Additionally, theoretical models often point to promising new directions for empirical research, even if these models do not provide immediately testable predictions (see below). When empirical results run counter to theoretical expectations, theorists and empiricists have an opportunity to discover unknown or underappreciated phenomena with potentially important consequences.

**Box 1. A Critical Connection—Assumptions**

Although the steps between assumptions and predictions in proof-of-concept models do not need to be empirically tested, empirical support is essential to ensure that key assumptions of mathematical models are biologically realistic. The process of matching assumptions to data is a two-way street; if a model demonstrates that a certain assumption is very important, it should motivate empirical work to see if it is met. Importantly, however, not all assumptions must be fully realistic for a model to inform our understanding of the natural world.

We can group assumptions into three general categories (with some overlap between them): we name these 1) critical, 2) exploratory, and 3) logistical. Critical assumptions are those that are integral to the hypothesis, analogous to the factors that an empirical scientist varies in an experiment (they would be part of the purple “hypothesis” box of Figure 1). These assumptions are crucial in order to properly test the verbal model; if they do not match the intent of the verbal hypothesis, then the mathematical model is not a true test of the verbal one. To illustrate this category of assumptions (and those below), consider the mathematical model by Rice [35], which tests the verbal model that “antagonistic selection between the sexes can maintain sexual dimorphism.” In this model, assumptions that fall into the critical category are that (i) antagonistic selection at a locus results in higher fitness for alternate alleles in each sex, and (ii) sexual dimorphism results from a polymorphism between these alleles. If critical assumptions cannot be supported by underlying data or observation, and are therefore biologically unrealistic, then the entire modeling exercise is devoid of biological meaning [36].

The second category, exploratory assumptions, may be important to vary and test, but are not at the core of the verbal hypothesis. These assumptions are analogous to factors that an empiricist wishes to control for, but that are not the primary variables. Examining the effects of these assumptions may give new insights and breadth to our understanding of a biological phenomenon. (These assumptions, and those below, might best fit in the blue “assumptions” box of Figure 1C.) Returning to Rice's [35] model of sexual dimorphism, two exploratory assumptions are the dominance relationship between the alleles under antagonistic selection and whether the locus is autosomal or sex linked. Analysis of the model shows that dominance does not affect the conditions for sexual dimorphism when the locus is autosomal, but it does when the locus is sex linked.

Finally, every mathematical modeling exercise requires that logistical assumptions be made. These assumptions are partly necessary for tractability. Additionally, proof-of-concept models in evolutionary biology, as in other fields, are not meant to replicate the real world; their purpose instead is to identify the effects of certain assumptions (critical and exploratory ones) by isolating them and placing them in a simplified and abstract context. A key to creating a meaningful model is to be certain that logistical assumptions made to reduce complexity do not qualitatively alter the model's results. In many cases, theoreticians know enough about the effects of an assumption to be able to make it safely. In Rice's [35] sexual dimorphism example, the logistical assumptions include random mating, infinitely large population size, and nonoverlapping generations. These are common and well-understood assumptions in many population genetic models. In other cases, the robustness of logistical assumptions must be tested in a specific model to understand their effects in that context. Because assumptions in mathematical models are explicit, potential limitations in applicability caused by the remaining assumptions can be identified; it is important that modelers acknowledge the potential effects of relaxing these assumptions to make these issues more transparent. As with the other categories of assumptions above, logistical assumptions have an analogy in empirical work; many experiments are conducted in lab environments, or under altered field conditions, with the same purpose of reducing biological complexity to pinpoint specific effects.

Much of the doubt about the applicability of models may stem from a mistrust of the effects of logistical assumptions. It is the responsibility of the theoretician to make his or her knowledge of the robustness of these assumptions transparent to the reader; it may not always be obvious which assumptions are critical versus logistical, and whether the effects of the latter are known. It is likewise the responsibility of the empirically-minded reader to approach models with the same open mind that he or she would an experiment in an artificial setting, rather than immediately dismiss them because of the presence of logistical assumptions.

**Box 2. The Complete Picture—Testing Predictions**

The predictions of some proof-of-concept models can be evaluated empirically. These tests are not “tests of the model”; the model is correct in that its predictions follow mathematically from its assumptions. They are, though, tests of the relevance or applicability of the model to empirical systems, and in that sense another way of testing whether the assumptions of the model are met in nature (i.e., an indirect test of the assumptions).

A well-known example of an empirical test of theoretically-derived predictions arises in local mate competition theory, which makes predictions about the sex ratio females should produce in their offspring in order to maximize fitness in structured populations, based on the intensity of local competition for mates [37]. These predictions have been assessed, for example, using experimental evolution in spider mites (*Tetranychus urticae*) [38]. The predictions of other evolutionary models might be best suited to comparative tests rather than tests in a single system. For example, inclusive fitness models suggest that, all else being equal, cooperation will be most likely to evolve within groups of close kin [6]. In support of this idea, comparative analyses suggest that mating with a single male (monandry), rather than polyandry, was the ancestral state for eusocial hymenoptera, meaning that this extreme form of cooperation arose within groups of full siblings [39].

In other cases, comparative data might be very difficult to collect. Theoretical models, for example, have demonstrated that speciation is greatly facilitated if isolating mechanisms that occur before and after mating are controlled by the same genes (e.g., are pleiotropic) [40]. While this condition is found in an increasing number of case studies [41], each case requires manipulative tests of selection and/or identification of specific genes, so that a rigorous comparative test of how often such pleiotropy is involved in speciation remains far in the future.

Proof-of-concept models can both bring to light hidden assumptions present in verbal models and generate counterintuitive predictions. When a verbal model is converted into a mathematical one, casual or implicit assumptions must be made explicit; in doing so, any unintended assumptions are revealed. Once these hidden assumptions are altered or removed, the predicted outcomes and resulting inferences of the formal model may differ from, or even contradict, those of the verbal model (Box 3). This benefit of mathematical models has brought clarity and transparency to virtually all fields of evolutionary biology. Additionally, in spite of their abstract simplicity, proof-of-concept models, much like simple, elegant experiments, have the capacity to surprise. Even formalizations of seemingly straightforward verbal models can yield outcomes that are unanticipated using a verbal chain of logic (Box 4). Proof-of-concept models thus have the ability both to reinforce the foundations of evolutionary explanations and to advance the field by introducing new predictions.

**Box 3. Uncovering Hidden Assumptions**

A striking example of the utility of mathematical models comes from the literature on the evolution of indiscriminate altruism (the provision of benefits to others, at a cost to oneself, without discriminating between partners who cooperate and partners who do not). Hamilton [6] proposed that indiscriminate altruism can evolve in a population if individuals are more likely to interact with kin. He also suggested that population viscosity—the limited dispersal of individuals from their birthplace—can increase the probability of interacting with kin. For a long time after Hamilton's original work, it was assumed, often without any explicit justification, that limited dispersal alone could facilitate the evolution of altruism [42]. A simple mathematical model by Taylor [43], however, showed that population viscosity alone cannot facilitate the evolution of altruism, because the benefits of close proximity to kin are exactly balanced by the costs of competition with those kin. Taylor's model revealed the importance of kin competition and clarified that additional assumptions about life history, such as age structure and the timing of dispersal relative to reproduction, are required for population viscosity to promote (or even inhibit) the evolution of altruism.

**Box 4. A Proof-of-Concept Model Finds a Flaw and Introduces a New Twist**

In stalk-eyed flies, males' exaggerated eyestalks play two roles in sexual selection: they are used in male–male competition and are the object of female choice. Researchers noticed that generations of experimental selection for less exaggerated eyestalks resulted in males that fathered proportionally fewer sons than expected [44]. Both verbal intuition and preliminary evidence led the research group to propose that females preferred males with long eyestalks because this exaggerated trait resided on a Y chromosome that was resistant to an X chromosome driver with biased transmission [45]. However, a proof-of-concept model highlighted the flawed logic of this verbal model; the mathematical model showed that females choosing to mate with males bearing a drive-resistant Y chromosome (as putatively indicated by long eyestalks) would have lower fitness than nonchoosy females, and therefore this preference would not evolve [46]. In contrast, female choice for long eyestalks could be favored if long eyestalks were genetically associated with a nondriving allele at the (X-linked) drive locus [46], so long as the eyestalk-length and drive loci were tightly linked [47]. These proof-of-concept models provided a new direction for empirical work, leading to the collection of new evidence demonstrating that the X-driver is linked to the eyestalk-length locus by an inversion [48], with the nondriver and long eyestalk in coupling phase (i.e., on the same haplotype).

## Investigating Evolutionary Puzzles through Proof-of-Concept Modeling

Proof-of-concept models have proven to be an essential tool for investigating some of the classic and most enduring puzzles in the study of evolutionary biology, such as “why is there sex?” and “how do new species originate?” These areas of research remain highly active in part because the relevant time scales are long and the processes are intricate. They represent excellent examples of topics in which mathematical approaches allow investigators to explore the effects of biologically complex factors that are difficult or impossible to manipulate experimentally.

### Why Is There Sex?

A century after Darwin [25] published his comprehensive treatment of sexual reproduction, John Maynard Smith [26] used a simple mathematical formalization to identify a biological paradox: why is sexual reproduction ubiquitous, given that asexual organisms can reproduce at a higher rate than sexual ones by not producing males (the “2-fold cost of sex”)? Increased genetic variation resulting from sexual reproduction is widely thought to counteract this cost, but simple proof-of-concept models quickly revealed both a flaw in this verbal logic and an unexpected outcome: sex need not increase variation, and even when it does, the increased variation need not increase fitness [27]. Subsequent theoretical work has illuminated many factors that facilitate the evolution and maintenance of sex. Otto and Nuismer [28], for example, used a population genetic model to examine the effects on the evolution of sex of antagonistic interactions between species. Such interactions were long thought to facilitate the evolution of sex [29],[30]. They found, however, that these interactions only select for sex under particular circumstances that are probably relatively rare. Although these predictions might be difficult to test empirically, their implications are important for our conceptual understanding of the evolution of sex.

### How Do New Species Originate?

Speciation is another research area that has benefitted from extensive proof-of-concept modeling. Even under the conditions most unfavorable to speciation (e.g., continuous contact between individuals from diverging types), one can weave plausible-sounding verbal speciation scenarios [22]. Verbal models, however, can easily underestimate the strength of biological factors that maintain species cohesion (e.g., gene flow and genetic constraints). Mathematical models have allowed scientists to explicitly outline the parameter space in which speciation can and cannot occur, highlighting many critical determinants of the speciation process that were previously unrecognized [31]. Felsenstein [32], for example, revolutionized our understanding of the difficulties of speciation with gene flow by using a proof-of-concept model to identify hitherto unconsidered genetic constraints. Speciation models in general have made it clear that the devil is in the details; there are many important biological conditions that combine to determine whether speciation is more or less likely to occur. Because speciation is exceedingly difficult to replicate experimentally, theoretical developments such as these have been particularly valuable.

### Pitfalls and Promise

Although mathematical models are potentially enlightening, they share with experimental tests the danger of possible overinterpretation. Mathematical models can clearly outline the parameter space in which an evolutionary phenomenon such as speciation or the evolution of sex can occur under certain assumptions, but is this space “big” or “little”? As with any scientific study, the impression that a model leaves can be misleading, either through faults in the presentation or improper citation in subsequent literature.

Overgeneralization from what a model actually investigates, and claims to investigate, is strikingly common in this age when time for reading is short [33], and this problem is exacerbated when the presentation is not accessible to readers with a more limited background in theoretical analysis [34]. Indeed, these problems, universal to many fields of science, introduce the greatest potential for error in the conclusions that the research community draws from evolutionary theory.

We follow this word of caution with a final positive thought: in addition to the roles of mathematical models in testing verbal logic, the ability of theory to circumvent practical obstructions of experimental tractability in order to tackle virtually any problem is a benefit that should not be underestimated. Science is a quest for knowledge, and if a problem is, at least currently, empirically intractable, it is very unsatisfactory to collectively throw up our hands and accept ignorance. Surely it is far better, in such cases, to use mathematical models to explore how evolution might have proceeded, illuminating the conditions under which certain evolutionary paths are possible.
